# Short‐term outcomes in children following emergency department visits for minor injuries sustained at home

**DOI:** 10.1186/s40621-021-00307-z

**Published:** 2021-04-26

**Authors:** Matthew J Molloy, Wendy Shields, Molly W Stevens, Andrea C Gielen

**Affiliations:** 1 Department of Pediatrics, Johns Hopkins University School of Medicine, Baltimore, USA; 2 Present affiliation: Cincinnati Children’s Hospital Medical Center, 3333 Burnet Ave, MLC 9016, OH 45229 Cincinnati, USA; 3 Department of Health Policy and Management, Johns Hopkins Bloomberg School of Public Health, Johns Hopkins Center for Injury Research and Policy, Baltimore, USA; 4 Department of Surgery, Division of Emergency Medicine, University of Vermont Larner College of Medicine, Burlington, USA; 5 Department of Health, Behavior, and Society, Johns Hopkins Bloomberg School of Public Health, Johns Hopkins Center for Injury Research and Policy, Baltimore, USA

**Keywords:** Minor injury, Injury outcomes, Pediatric injury, Pediatric burn, Pediatric emergency medicine, Substandard housing

## Abstract

**Background:**

Minor injuries are very common in the pediatric population and often occur in the home environment. Despite its prevalence, little is known about outcomes in children following minor injury at home. Understanding the impact of these injuries on children and their families is important for treatment, prevention, and policy. The objectives of our study were (1) To describe the distribution of short-term outcomes following pediatric minor injuries sustained at home and (2) To explore the relationship of injury type and patient and household demographics with these outcomes.

**Methods:**

Children (*n* = 102) aged 0–7 years with a minor injury sustained at home were recruited in an urban pediatric emergency department as part of the Child Housing Assessment for a Safer Environment (CHASE) observational study. Each patient had a home visit following the emergency department visit, where five parent-reported outcomes were assessed. Relationships were explored with logistic regression.

**Results:**

The most common type of injury was soft tissue (57.8 %). 13.2 % of children experienced ≥ 7 days of pain, 21.6 % experienced ≥ 7 days of abnormal activity, 8.9 % missed ≥ 5 days of school, 17.8 % of families experienced ≥ 7 days of disruption, and 9.1 % of parents missed ≥ 5 days of work. Families reported a total of 120 missed school days and 120 missed work days. Children who sustained a burn had higher odds of experiencing pain (OR 6.97), abnormal activity (OR 8.01), and missing school (OR 8.71). The parents of children who sustained a burn had higher odds of missing work (OR 14.97).

**Conclusions:**

Families of children suffering a minor injury at home reported prolonged pain and changes in activity as well as significant school and work loss. In this cohort, burns were more likely than other minor injuries to have these negative short-term outcomes reported and represent an important target for interventions. The impact of these injuries on missed school and disruption of parental work warrants further consideration.

## Background

Injury is the leading cause of morbidity and mortality among children worldwide, with children < 20 years of age in the United States experiencing an annual nonfatal injury rate of 11,272 per 100,000 [1]. Among children in this age group, the Centers for Disease Control and Prevention (CDC) estimates that 9.2 million children visit the emergency department annually for an unintentional injury, representing 25–40 % of all pediatric emergency department (PED) visits [1, 2]. Of children seeking treatment in the PED, approximately 75–90 % do not require admission to the hospital, and are thus classified as minor injury [3, 4].

The home environment represents the most common location of minor injury in young children [5–8]. Children < 5 years are at highest risk of these injuries, with falls representing the most common mechanism of injury [5, 9, 10]. In the US, injuries sustained at home account for an average of 4 million PED visits per year, representing approximately 40 % of PED visits for unintentional injury in children < 20 years [5].

Despite its prevalence, very little data have been reported about the outcomes experienced by children following minor injury. A study of 334 children ages 2 to 18 years with minor injury found that 16 % of children had 7 or more days of pain, 35 % of children had 7 or more days of abnormal activity, and 7 % of children missed 5 or more days of school. Parents missed 7 or more days of work in 13 % of cases [11]. There is an absence of data about short-term outcomes specific to children that sustain a minor injury at home.

It has long been known that injuries disproportionately affect minority groups and socioeconomically disadvantaged children [12, 13], and disparities in housing quality and a child’s home environment may contribute to this increased risk of injury. Low housing quality itself can be a barrier to child safety [14]. The CDC has noted the link between substandard housing and important health problems, including asthma, lead, and injuries [15]. The relationship between housing quality and outcomes following minor injury in the home environment has not been described.

Our study seeks to examine the short-term outcomes among an urban population of young children who sustain a minor injury in their home environment, addressing the gap in knowledge surrounding this most common reason for children seeking treatment in the PED for an injury. We also seek to explore the relationship of these short-term outcomes to patient and household variables to better understand who is at highest risk. The specific objectives of our study are to: (1) describe the distribution of short-term outcomes following pediatric minor injuries sustained at home in an urban population, and (2) explore the relationship of injury type and patient demographics, including household variables, to short-term outcomes of children injured at home. We hypothesize that young children injured at home experience short-term outcomes similar to minor injuries more generally and that certain injury types are associated with worse short-term outcomes.

## Methods

### Study Population

Participants were recruited as part of the Child Housing Assessment for a Safer Environment (CHASE) study [16], a study aimed at addressing the gap between pediatric housing-related injuries and housing policy. Children with minor injury, defined as an injury that was treated and released consistent with CDC disposition criteria, were identified in the PED of a large urban medical center from January 2012 to December 2012. The inclusion criteria were: (1) child was aged from birth to 7 years, (2) child had a PED visit that was not a follow-up visit, (3) child was discharged home, (4) home address was in Baltimore City or County, (5) parent/guardian was English-speaking, (6) child lived with the parent/guardian most or all of the time, and (7) the injury occurred in the home where the child lives most of the time. Enrollment was restricted to English-speaking families because study data collectors were not equipped to work with non-English-speaking families. A child was excluded from the study if suspicion of non-accidental trauma was noted on the child’s medical record.

Cases of unintentional injury that occurred at home (e.g., not motor vehicle-related, not playground-related, etc.) were identified by examining the PED tracking system prior to subject recruitment. Recruitment occurred both in person and following the visit via mail and telephone. Study team members used the PED notes to extract the child’s age and gender and to classify the injury mechanism and type. The injury mechanism was classified as one of five categorical variables consistent with CDC classification: fall, burn (including chemical burn), cut/pierce, struck by/against, and inhalation injury (including carbon monoxide poisoning). The injury type was classified as one of five categorical variables consistent with CDC classification and grouped for purposes of analysis: soft tissue injury (superficial, contusion, or open wound), fracture/sprain, minor head injury, burn, and inhalation.

A one-time home visit by two trained study data collectors was completed within one to eight weeks following the PED visit when the parent/guardian who accompanied the child to the PED was available. The visit included an interview with the parent/guardian. Parents were informed about the study at the time of initial contact and written informed consent was obtained from the parent/guardian at the time of the home visit. This study was approved by the Johns Hopkins Bloomberg School of Public Health Institutional Review Board, IRB Number 00002381. The study protocol was developed in collaboration with a community advisory board and with attention to guidance provided in the Institute of Medicine Report entitled *Ethical Considerations for Research on Housing-Related Health Hazards Involving Children* [17].

### Patient and Household Variables

The in-home parent interview assessed demographic information, including parent self-reported race and ethnicity, parent education level, and estimated household income. The home was classified into one of four housing categories: (1) row house, townhouse, or duplex, (2) detached single family home, (3) apartment in a house, (4) apartment in a building. For purposes of this analysis, housing type was dichotomized to house (categories 1&2) and apartment (categories 3&4). We classified families as being above or below the Federal Poverty Level (FPL) based on the reported household income and the number of people in the household.

A home inspection was conducted during the visit and the quality of the house was assessed using the United States Department of Housing and Urban Development (HUD)’s Housing Quality Standards (HQS) checklist, the inspection form used to qualify a home for the Housing Choice Voucher Program [18]. In order to pass the inspection, all standards had to pass (i.e., any failed standard would constitute a failed inspection). Houses that failed one or more of the quality standards were classified as “substandard”. Families were asked about any changes to the home environment since the injury and are reported elsewhere [19].

### Patient and family outcomes

Short-term parent-reported patient and family outcomes, specified as resulting from the injury, were assessed as part of the interview during the home visit. The short-term outcomes assessed were: (1) days of pain following PED visit, (2) days of abnormal activity following PED visit, (3) days of school or daycare missed following PED visit (for children who attended school or daycare), (4) days of work missed by parent following PED visit (for parents who were working), and (5) days of family disruption following PED visit. Abnormal activity was assessed by asking the parent guardian how many days it took for their child to return to normal in the following domains: (1) activity outside the home, (2) movement, walking, or climbing stairs, (3) sleeping or eating, (4) experiencing tiredness or fatigue. Days of abnormal activity was defined as the longest duration of abnormal activity reported in any of the four domains. Family disruption was assessed by asking the parent/guardian “How many days did it take for family activities or routines to return to normal?”. These five outcomes have been used in previous literature [3, 11]. For purposes of analysis, outcomes were dichotomized as favorable (< 1 week) or poor (lasting ≥ 1 week). This one-week cutoff has been used in previous literature assessing short-term injury outcomes [11], and was also chosen because some home visits occurred as early as seven days following discharge from the PED. A cutoff of five days was used for days of school and work missed to reflect the school/work week.

### Statistical analysis

All statistical analyses were performed using Stata statistical software version 13.1 (StataCorp LP, College Station, TX). Descriptive statistics of injured children and the distribution of injury mechanism and injury type were tabulated. The distributions of short-term outcomes over time were displayed using Kaplan-Meier curves.

The relationship between the dichotomous short-term outcomes and demographic and household variables was explored using multiple logistic regression with injury type, child sex, child age, parent race, poverty status, and housing quality as covariates. Adjusted odds ratios, 95 % confidence intervals, and Wald statistics were calculated for each covariate. Injury type was used in the analyses rather than injury mechanism because the injury itself, and not its mechanism, would more logically influence outcomes (e.g., a fracture might be expected to have different outcomes than a head injury, though both could be sustained from a fall). Soft tissue injuries were used as the reference category to examine the influence of injury type on poor outcomes because they were the most common type of injury.

Collinearity for the logistic regression analyses was checked by performing multiple linear regression analyses to calculate the variance inflation factors, which were below 2.0 for all covariates included in the models. The risk of overfitting was controlled by using a ratio of approximately 1:10 for the number of explanatory variables and sample size.

## Results

### Patient and Household characteristics

 A total of 1023 families were invited to participate in the study; 104 children with minor unintentional injury were enrolled and had a home visit completed. Further details about the sample are available elsewhere [16]. Two children with inhalation injury, which were both carbon monoxide related, were excluded because of the unique nature of their injury, leaving 102 children in the analysis. Home visits and surveys were conducted 27 days following PED visit on average, with the earliest visit occurring after 7 days and the latest occurring after 57 days. More than two-thirds of home visits occurred within one month of ED visit.

Patient and family demographics and household variables are presented in Table 1. The average age of children was 2.88 years and approximately 60 % of the children were male. Over three-quarters of enrolled parents self-identified as black or African-American and nearly half (48 %) of patients lived in a household reported to be below the Federal Poverty Level. Nearly 80 % of children lived in a row house, townhouse, duplex, or detached home. Notably, 82 % of children lived in what was observed to be substandard housing.

**Table 1:** Baseline Patient and Household Characteristics

Demographic Variables	*N* = 102
**Sex (%)**	
Male Female	60 (58.8) 42 (41.2)
**Race (%)**	
Black or African American White or Caucasian Other	77 (75.5) 16 (15.7) 9 (8.8)
**Age – Years**	
Mean (SD)	2.88 (1.85)
**Reported Family Income (%)**	
Less than $5,000 $5,000 to $14,999 $15,000 to $24,999 $25,000 to $34,999 $35,000 or more I don’t know	20 (19.6) 18 (17.7) 14 (13.7) 12 (11.8) 28 (27.5) 10 (9.8)
**Below Federal Poverty Level**^**a**^ **(%)**	
No Yes I don’t know	39 (38.2) 49 (48.0) 14 (13.7)
**Parent’s Education Level (%)**	
Less than High School Completed High School or GED Some college Completed college or more	26 (25.5) 25 (24.5) 31 (30.4) 20 (19.6)
**Parent’s Employment Status**	
Employed Not Employed	99 (97.1) 3 (2.9)
**Child’s Schooling Status**	
Child in school or daycare Child not in school and not in daycare	79 (77.5) 23 (22.5)
**Housing Type (%)**	
House Apartment	81 (79.4) 21 (20.6)
**Substandard Housing**^**b**^ **(%)**	
No Yes	18 (18.2) 81 (81.8)

^a^Federal Poverty Level calculation based on reported family income and family size

^b^Substandard Housing designation based on HUD’s Housing Quality Standard assessment. Housing failing any quality standard received a designation of “substandard”

### Distribution of Injury

The distribution of injury mechanism and injury type is presented in Table 2. Falls accounted for over half of the injury mechanisms in this study (54.9 %) and soft tissue injuries accounted for over half of the injury types in this study (57.8 %). Neither injury mechanism nor injury type differed significantly by sex, race, poverty status, housing type, or housing quality. 

**Table 2:** Injury Mechanism and Injury Type.

Injury Variable	N = 102
**Injury Mechanism (%)**	
Fall Cut/Pierce Struck By/Against Burn	56 (54.9) 9 (8.8) 21 (20.6) 16 (15.7)
**Injury Type (%)**	
Soft Tissue Injury Fracture/Sprain Minor Head Injury Burn	59 (57.8) 11 (10.8) 16 (15.7) 16 (15.7)

### Short-Term clinical outcomes following Minor Injury

**Fig. 1:**
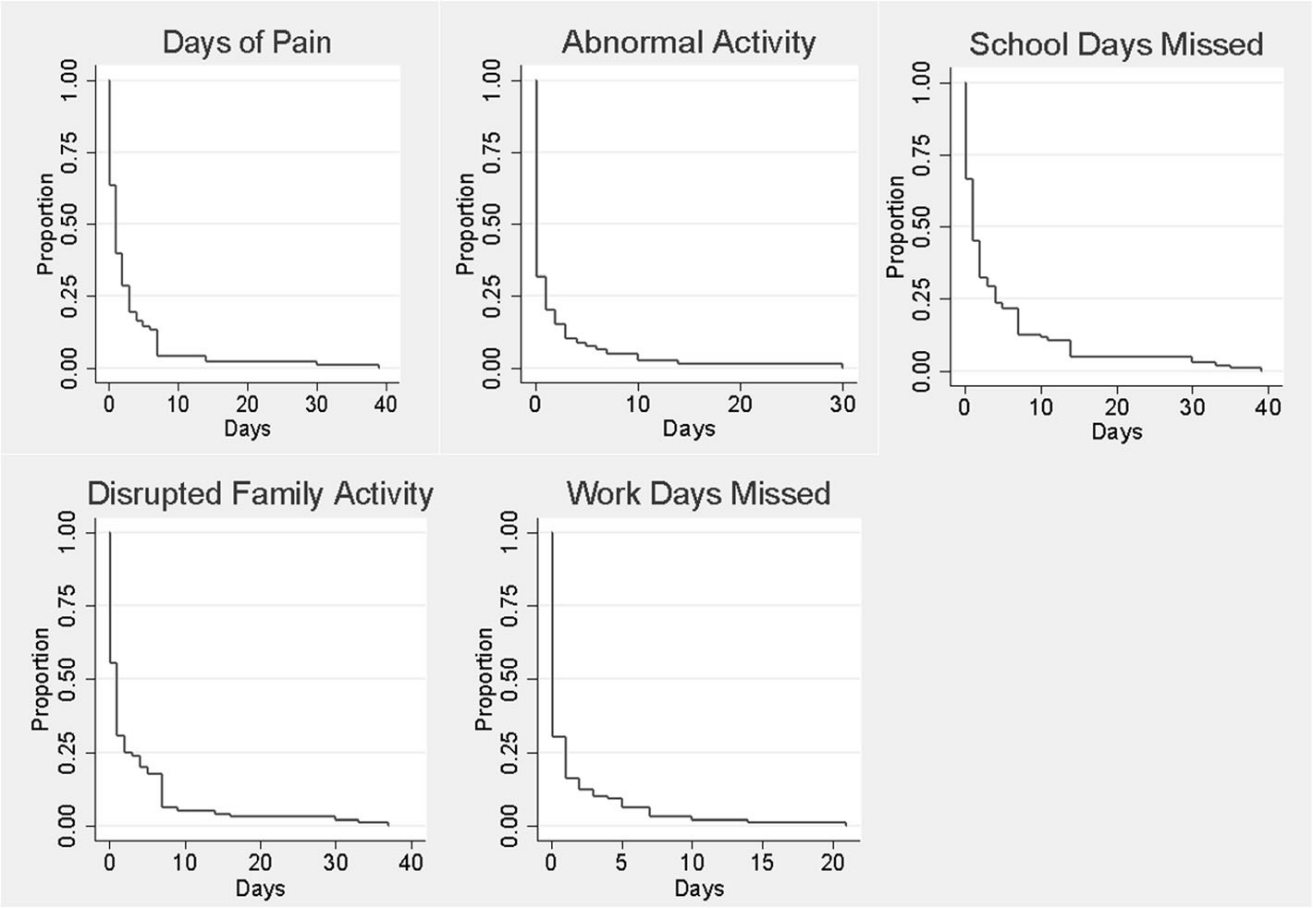
Kaplan-Meier curves of short-term outcomes following pediatric minor injuries sustained at home. Days of pain reported for 98 children. Abnormal activity reported for 102 children. School days missed reported for 79 children. Disrupted family activity reported for 101 children. Work days missed reported for 99 parents.

Children experienced a median of one day of pain following PED visit (range 0 to 39 days). 13 % of children experienced seven or more days of pain. Children experienced a median of one day of abnormal activity following PED visit (range 0 to 39 days). 22 % of children experienced seven or more days of abnormal activity. Children who attended school or daycare missed a median of zero days of school following PED visit (range 0 to 30 days), with a total of 120 days missed among the 79 children. Only 8.8 % of these 79 children missed five or more days of school. Families reported a median of one day of disrupted activity following a child’s PED visit (range 0 to 37 days). A total of 17.8 % of the families experienced seven or more days of disrupted activity. Working parents reported missing a median of zero days of work (range 0 to 21 days), with a total of 120 workdays missed among 99 participants who were employed. 9 % of those 99 parents missed five or more days of work. The Kaplan-Meier curves displaying the distribution of the short-term outcomes are displayed in Fig. 1. The curves show rapid drops with long tails, demonstrating most children had few days of negative outcomes following minor injury with a small number of children experiencing a longer duration of negative effects.

### Logistic Regression Analyses

The results of the multiple logistic regression models for poor outcomes are displayed in Table 3. Injury type was statistically associated with poor outcomes for pain (Wald test *p* = 0.041) and abnormal activity (Wald test *p* = 0.030). Holding other variables constant, compared to children who sustained a soft tissue injury, children who sustained a burn had a seven times increased odds of experiencing a week or more of pain (OR 6.97, CI 1.53–31.83, *p* = 0.012), an eight times increased odds of experiencing a week or more of abnormal activity (OR 8.01, CI 2.03–31.65, *p* = 0.003), nearly a nine times increased odds of missing 5 or more days of school (OR 8.71, CI 1.11–68.05, *p* = 0.039), and a nearly fifteen-fold increase in the odds of parents missing 5 or more days of work (OR 14.97, CI 2.13-105.01, *p* = 0.006). Other demographic and housing variables, including sex, age, race, poverty status, and substandard housing status, were not associated with any of the outcomes, nor were any of the other types of injuries when compared to soft tissue injuries (Table 3).

**Table 3:** Multiple Logistic Regression Analyses of Poor Outcomes Following Minor Injury

	**Outcome Variable 1:** **7+ days of pain (*N*=98)**			**Outcome Variable 2:** **7+ days of abnormal activity (*N*=102)**		
**Covariates**	**AOR**	**95% CI**	***p*-value**	**AOR**	**95% CI**	***p*-value**
Injury Type			0.041^*, **^			0.030^*, **^
Soft Tissue	REF	REF		REF	REF	
Fracture/Sprain	1.58	0.22-11.27	0.650	1.31	0.21-8.04	0.771
Minor Head Injury	N/A	N/A	N/A	1.91	0.36-10.01	0.443
Burn	6.97	1.53-31.83	0.012*	8.01	2.03-31.65	0.003*
Sex (Male=REF)	2.48	0.62-9.77	0.195	1.71	0.58-5.07	0.334
Child Age (years)	1.42	0.98-2.09	0.064	1.34	0.98-1.83	0.064
Parent Race			0.813^**^			0.777^**^
Black	REF	REF		REF	REF	
White	1.98	0.24-15.72	0.520	1.25	0.26-6.02	0.777
Other	1.22	0.05-28.94	0.903	N/A	N/A	N/A
Poverty			0.620^**^			0.720^**^
Above FPL^a^	REF	REF		REF	REF	
Below FPL	2.23	0.39-12.78	0.366	0.60	0.17-2.13	0.428
I Don’t Know	2.62	0.22-31.18	0.444	0.85	0.12-6.22	0.871
Substandard Housing						
No	REF	REF		REF	REF	
Yes	0.89	0.15-5.27	0.898	1.69	0.34-8.35	0.518
	**Outcome Variable 3: 5+ school days missed (*N*=79)**			**Outcome Variable 4:** **7+ days disrupted family activity** **(*N*=101)**		
**Covariates**	**AOR**	**95% CI**	***p*-value**	**AOR**	**95% CI**	***p*-value**
Injury Type			0.118^**^			0.584^**^
Soft Tissue	REF	REF		REF	REF	
Fracture/Sprain	N/A	N/A	N/A	1.05	0.17-6.67	0.956
Minor Head Injury	2.78	0.19-40.06	0.454	1.13	0.18-7.07	0.895
Burn	8.71	1.11-68.05	0.039*	2.62	0.66-10.42	0.173
Sex (Male=REF)	0.88	0.12-6.48	0.901	2.22	0.71-6.92	0.168
Child Age (years)	1.46	0.85-2.49	0.172	1.30	0.95-1.79	0.101
Parent Race			0.847^**^			0.790^**^
Black	REF	REF		REF	REF	
White	1.31	0.08-21.25	0.778	1.26	0.23-6.96	0.790
Other	N/A	N/A	N/A	N/A	N/A	N/A
Poverty			0.778^**^			0.684^**^
Above FPL^a^	REF	REF		REF	REF	
Below FPL	1.31	0.19-9.02	0.778	1.54	0.40-5.90	0.529
I Don’t Know	N/A	N/A	N/A	0.69	0.06-7.86	0.767
Substandard Housing						
No	REF	REF		REF	REF	
Yes	N/A	N/A	N/A	0.61	0.14-2.71	0.515
	**Outcome Variable 5:** **5+ days of missed work (*N*=99)**					
**Covariates**	**AOR**	**95% CI**	***p*-value**			
Injury Type			0.050^**^			
Soft Tissue	REF	REF				
Fracture/Sprain	2.05	0.14-28.69	0.595			
Minor Head Injury	2.58	0.16-41.52	0.503			
Burn	14.97	2.13-105.01	0.006*			
Sex (Male=REF)	1.42	0.27-7.51	0.680			
Child Age (years)	1.29	0.83-2.01	0.252			
Parent Race			0.640^**^			
Black	REF	REF				
White	N/A	N/A	N/A			
Other	2.05	0.10-41.26	0.640			
Poverty			0.704^**^			
Above FPL^a^	REF	REF				
Below FPL	2.16	0.28-16.69	0.461			
I Don’t Know	0.97	0.04-23.79	0.986			
Substandard Housing						
No	REF	REF				
Yes	1.12	0.12-10.41	0.918			

*AOR* Adjusted odds ration; *CI* confidence interval; *REF* reference. Note that “N/A” indicates that no poor outcomes occurred in that variable.

^a^ FPL = Federal Poverty Level

* Denotes significant *p*-value (< 0.05)

^**^ Represents overall *p*-value for covariate

## Discussion

Our study fills an important gap in our understanding of the morbidity of minor injuries in young children that occur in the home environment. The families of injured children in this study reported notable morbidity following a PED visit. 13 % experienced a week or more of pain, 22 % experienced a week or more of abnormal activity, and 9 % of children who attended daycare or school missed 5 or more days. 18 % of families experienced a week or more of disrupted activity and 9 % of working parents missed 5 or more days of work. These short-term outcomes are similar to those reported by an older cohort of children with minor injury [11].

The morbidity experienced by patients and their families following a minor injury is substantial, especially when scaled to the number of children experiencing minor injury in the U.S. on an annual basis. In our cohort of only 102 injured children, there were 120 missed school days and 120 missed parent workdays. While this was not a completely representative sample of injuries or families, extrapolating these findings to national data would mean millions of days of school missed by children and millions of days of work missed by parents every year due to minor injuries sustained at home. In addition to its health consequences, minor injury in children could have negative impacts on child education and parental productivity. Low-income families are disproportionately affected when their children are sick or injured because they are less likely to have access to paid sick leave [20, 21].

None of the demographic or housing variables were associated with poor outcomes, although injury type was. Specifically, children who sustained a burn had higher odds of experiencing poor outcomes for pain, abnormal activity, missed school, and missed work by parent. Pain associated with burns in children is difficult to treat [22]. The costs associated with parents missing work when their children suffer a minor burn add to the significant costs to the healthcare system from inpatient care of pediatric burns [23]. Burn prevention could represent an important target to reduce the overall morbidity associated with minor home injuries in children.

In this cohort of children, 82 % lived in substandard housing. Policy efforts to improve access to quality housing include programs like the Housing Choice Voucher Program (“Section 8” housing) [24]. Low-income families are provided with vouchers to pay for housing that meets minimum Housing Quality Standards, yet there has been no research into how this program and other housing policies could be improved to prevent injuries in children. Given the association between poor housing quality and increased presence of burn risks [25], and that families living in substandard housing face barriers to making their homes safer [14, 26], addressing the burn risks of children living in substandard housing represents an important first step to mitigating the impact of negative outcomes following minor home injury. In this sample, most of the burns were scald burns from tap water or hot liquid. Scald risk could be addressed by updating the Housing Quality Standards required of homes participating in the Housing Choice Voucher Program to require testing, adjusting, and retesting of homes’ water temperatures as part of home inspections, to regulate that water temperatures are 120 degrees Fahrenheit or less, and to regulate that water heaters or household plumbing include a mixing valve [27, 28].

Our study has a number of limitations. This analysis is a secondary analysis of a larger study that was not powered or designed specifically to assess the short-term outcomes in question. This study represents a relatively small convenience sample of children recruited from one large urban academic center and living in the same city, with a large percentage living in what was observed to be substandard housing. Only English-speaking families were included. These factors limit generalizability. While injury distribution and poor outcome frequencies were consistent with previous literature [1, 9, 11], our results should not be generalized to all populations of young children experiencing minor injury in their homes and may not have been a representative sample of children at our center. Outcomes were assessed using retrospective parental report, which may not accurately reflect outcomes and might introduce reporting bias. In addition, the length of time between ED visit and home visit varied between participants, which impacted our ability to assess duration of outcomes beyond one week in some children. Though all the injuries were minor enough for the patients to be discharged home, there was likely a distribution in the severity of these injuries that was not captured and may impact short-term outcomes. Nevertheless, this study represents an important contribution to understanding the absolute and relative impact of injuries that occur in a child’s home environment and identifies opportunities for preventive interventions, especially in the area of burns. These findings are all the more important in the context of the increased amount of time that families are spending in their homes during the COVID-19 pandemic and the potential for more injuries [29]. Our findings support the need for further focused and prospective study of outcomes following these injuries.

## Conclusions

Our study describes short-term outcomes following minor injuries sustained in the home environment and demonstrates considerable morbidity, particularly following a burn. We report important data on school absenteeism and disruption of parental work schedule that highlight the impact these injuries can have on a family, community, and national level. These data represent an important addition to our understanding of injury epidemiology and help to better contextualize the burden of commonly occurring minor injuries.

## Data Availability

The datasets used and analyzed during the current study are available from the corresponding author on reasonable request.

## Abbreviations

AOR: Adjusted odds ratio
CDC: Centers for Disease Control and Prevention
CI: Confidence interval
COVID-19: Coronavirus disease 2019
GED: General Education Development
HQS: Housing Quality Standards
HUD: United States Department of Housing and Urban Development
IRB: Institutional review board
OR: Odds ratio
PED: Pediatric emergency department
SD: Standard deviation
US: United States of America
